# MiR-196a2 and lung cancer in Chinese non-smoking females: a genetic association study and expression analysis

**DOI:** 10.18632/oncotarget.20174

**Published:** 2017-08-10

**Authors:** Zhihua Yin, Zhigang Cui, Yangwu Ren, Lingzi Xia, Hang Li, Baosen Zhou

**Affiliations:** ^1^ Department of Epidemiology, School of Public Health, China Medical University, Shenyang 110122, PR China; ^2^ Key Laboratory of Cancer Etiology and Intervention, University of Liaoning Province, Shenyang 110122, PR China; ^3^ School of Nursing, China Medical University, Shenyang 110122, China

**Keywords:** lung cancer, microRNA, single nucleotide polymorphism, genetic susceptibility, expression

## Abstract

**Background:**

The common polymorphism rs11614913 in miR-196a2 might be associated with lung cancer risk for non-smoking females in northeast China.

**Methods:**

The genotypes of rs11614913 in miR-196a2 were determined by a case-control study including 1003 patients with lung cancer and 1003 healthy controls. The tissues were detected to assess the miRNA expression. Secondary structures of miR-196a2 were predicted.

**Results:**

There was a significant association between miR-196a2 rs11614913 and lung cancer risk in Chinese non-smoking females. Individuals carrying TC or CC genotype had increased risk of lung cancer compared with TT genotype (adjusted risks were 1.63 and 1.67). The C allele was associated with a higher risk of lung cancer with a significant risk of 1.27. The similar significant results were also found in lung adenocarcinoma. There was a significant association between miR-196a2 expression and lung cancer risk (t=2.594, P=0.012). The relative expression of miR-196a2 was significantly higher for CC genotype comparing with the CT or TT genotype in tumor tissues (P values were all 0.003). The optimal free energies were different for T allele and C allele.

**Conclusions:**

The polymorphism rs11614913 in miR-196a2 may be associated with lung cancer risks in Chinese non-smoking females through affecting miR-196a2 expression and secondary structure.

## INTRODUCTION

Based on GLOBOCAN estimates, about 1.8 million new lung cancer cases occurred in 2012, accounting for about 13% of total cancer diagnoses [1]. Among females, lung cancer was the second leading cause of cancer death in less developed countries. Lung cancer rates in Chinese women (20.4 cases per 100,000 women) were higher than rates among women in some European countries despite a lower prevalence of smoking, suggesting that in addition to tobacco, there are other impact factors involved in the etiology of lung cancer, especially for women [1]. Molecular epidemiologic studies have shown that there were hundreds of genes involved in lung carcinogenesis and newly developed biomarkers such as noncoding small RNAs may lead to novel understanding in the molecular mechanisms of lung cancer [2]. MicroRNAs (miRNAs) are a class of small (about 21- to 24-nucleotide long) non-coding RNAs, which are considered to influence gene expression at the post transcriptional level. MiRNAs are considered to be tissue-specifically expressed and changes of their expression are suggested to be related to disease status [3-4]. More than half of miRNA genes located in cancer-associated genomic regions or in fragile sites so miRNAs might be involved in human cancers [5], acting as tumor suppressors or oncogenes [6]. MiRNA expression profiling revealed that most of them are differentially expressed in human lung cancer [7]. The molecular mechanism is still unclear, but transcriptional inhibition, genetic and epigenetic mechanisms, histone acetylation and deacetylation, or regulation of miRNA stability might be involved [8].

To understand the relationship between miRNAs and cancer, the presence of sequence variants in miRNA such as single nucleotide polymorphisms (SNPs) are the hot topic. Several lines of evidences indicate that SNPs in miRNA containing genomic regions may significantly affect the expression, production or of miRNAs [9-11]. The SNPs in miRNA sequences are concluded to influence expression, processing, secondary structure and/or target gene of miRNAs, and consequently modify cancer risk [9, 12-15]. Taken together, one SNP in a miRNA sequence could lead to a vital functional alteration and may be a putative cancer biomarker.

In recent years, our research group have focused on the association between SNPs in non-coding RNA genes and lung cancer risks, and the relationships between the eight SNPs in miRNAs and the risk of lung cancer for non-smoking females in northeast China have been firstly reported [16-18]. At first, the borderline significance was showed for rs11614913 in miR-196a2 in our published results [16]. Then we explored the gene-environment interaction among limited cases and controls with exposure data and found the significant results for rs11614913 in miR-196a2 [18]. A recent meta-analysis showed that the rs11614913 in miR-196a2 was likely to be associated with lung cancer risks [19]. However, in the association studies, the influence of this SNP on the expression of miR-196a2 is not always clarified. In the present study, more cases and controls have been added to verify the association between miR-196a2 rs11614913 polymorphism and lung cancer risk, but also its effects on miR-196a2 expression and secondary structure were also reported.

## RESULTS

In the present study, there are 1003 cases and 1003 controls, among which the results in 575 cases and 608 controls have been reported [16]. All individuals were nonsmoking females. The mean ages were 57.03±11.72 and 56.27±12.56 in cases and controls, which were identical (t=-1.413 and P=0.158). Among all lung cancer cases, 683 patients were adenocarcinoma, 174 were squamous cell carcinoma and 146 other types. The distributions of miR-196a2 rs11614913 polymorphism in the cases and controls were shown in Table 1. The frequencies of three genotypes in the controls were in agreement with that expected under the Hardy-Weinberg equilibrium (P = 0.830).

**Table 1:** The association of miR-196a2 polymorphism and lung cancer risk

SNP	Cases(%)	Controls(%)	OR (95%CI)	P value	OR (95%CI)^*^	P value
TT(ref)	196 (19.5)	286 (28.5)	1.00 (ref)		1.00 (ref)	
TC	555 (55.3)	496 (49.5)	1.63 (1.31-2.03)	<0.001	1.63 (1.31-2.03)	<0.001
CC	252 (25.1)	221 (22.0)	1.66 (1.29-2.15)	<0.001	1.67 (1.29-2.16)	<0.001
Dominant model TC+CC vs TT			1.64 (1.33-2.02)	<0.001	1.64 (1.33-2.02)	<0.001
Recessive model CC vs TT+TC			1.19 (0.97-1.46)	0.103	1.19 (0.97-1.47)	0.095
T allele(ref)	947 (47.2)	1068 (53.2)	1.00 (ref)	—		
C allele	1059 (52.8)	938 (46.8)	1.27 (1.13-1.44)	<0.001		

SNP, single nucleotide polymorphism; OR, odds ratio; CI, confidence interval.

^*^ORs were calculated by unconditional logistic regression and adjusted for age.

The association between miR-196a2 rs11614913 and lung cancer risk was shown in Table 1. Individuals carrying heterozygote (TC) or variant homozygote (CC) genotype had increased risk of lung cancer compared with the wildtype TT genotype (adjusted ORs were 1.63 and 1.67, 95%CIs were 1.31-2.03 and 1.29-2.16, P values were all <0.001). In the dominant model, we found that the combination of TC and CC genotypes was associated with a significantly high risk of lung cancer compared with TT genotype (adjusted OR=1.64, 95%CI=1.33-2.02, P<0.001). Allele comparison showed that the C allele was associated with a higher risk of lung cancer with a significant OR of 1.27 (95%CI=1.13-1.44, P<0.001). Further analyses were carried on by stratified analyses by pathological type of cancer and similar significant results were found in lung adenocarcinoma but not in squamous cell lung cancer (Table 2).

**Table 2:** The association of miR-196a2 polymorphism and lung cancer risk by cancer type

SNP	OR (95%CI)	P value	OR (95%CI)^*^	P value
**Adenocarcinoma**				
TT(ref)	1.00 (ref)		1.00 (ref)	
TC	1.85 (1.44-2.37)	<0.001	1.84 (1.43-2.37)	<0.001
CC	1.93 (1.44-2.58)	<0.001	1.94 (1.45-2.59)	<0.001
Dominant model TC+CC vs TT	1.87 (1.47-2.38)	<0.001	1.87 (1.47-2.38)	<0.001
Recessive model CC vs TT+TC	1.26 (1.00-1.58)	0.048	1.26 (1.01-1.58)	0.044
T allele(ref)	1.00 (ref)	—		
C allele	1.35 (1.18-1.55)	<0.001		
**Sqamous cell cancer**				
TT(ref)	1.00 (ref)		1.00 (ref)	
TC	1.47 (0.98-2.20)	0.066	1.46 (0.97-2.20)	0.069
CC	1.50 (0.94-2.41)	0.091	1.53 (0.95-2.46)	0.080
Dominant model TC+CC vs TT	1.48 (1.00-2.18)	0.049	1.48 (1.00-2.19)	0.048
Recessive model CC vs TT+TC	1.16 (0.80-1.69)	0.435	1.18 (0.81-1.72)	0.382
T allele(ref)	1.00 (ref)	—		
C allele	1.22 (0.97-1.53)	0.087		

SNP, single nucleotide polymorphism; OR, odds ratio; CI, confidence interval.

*ORs were calculated by unconditional logistic regression and adjusted for age.

**Figure 1:**
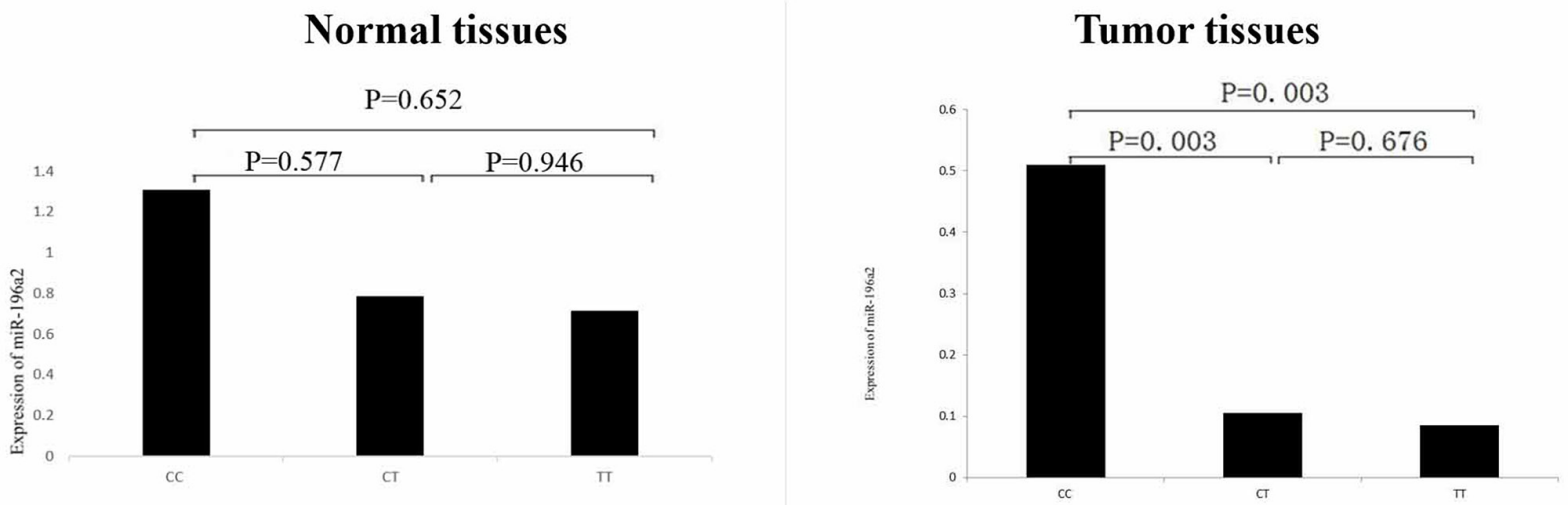
Comparison of expression of miR-196a2 in benign lung and lung cancer tissues stratified according to the three genotypes of rs11614913 Sequence variations in the miR-196a2 can influence its expression. The relative expression (2^-^ΔΔ^Ct^) of miR-196a2 to U6 was significantly different between the CC genotype and the CT or TT genotype.

The mean age of 60 individuals for quantitative real time RT-PCR was 55.9 with standard deviation of 1.41. There were 24 subjects with adenocarcinoma, 22 squamous cell cancer and 14 other types. The ΔΔCt values of the cases and controls were -1.84±2.81 and 0.00±2.86 and the difference was significant (t=2.594, P=0.012), suggesting that miR-196a2 expression was associated with lung cancer risk. To further identify the functional relevance of rs11614913, correlation between rs11614913 and the expression of miR-196a2 was performed. As shown in Figure 1, the relative expression of miR-196a2 to U6 (2^-^ΔΔ^Ct^) was significantly different when we compared the CC genotype with the CT or TT genotype (P values were all 0.003) in tumor tissues. However, there were no significant differences of miR-196a2 expression in normal tissues between different genotypes.

**Figure 2:**
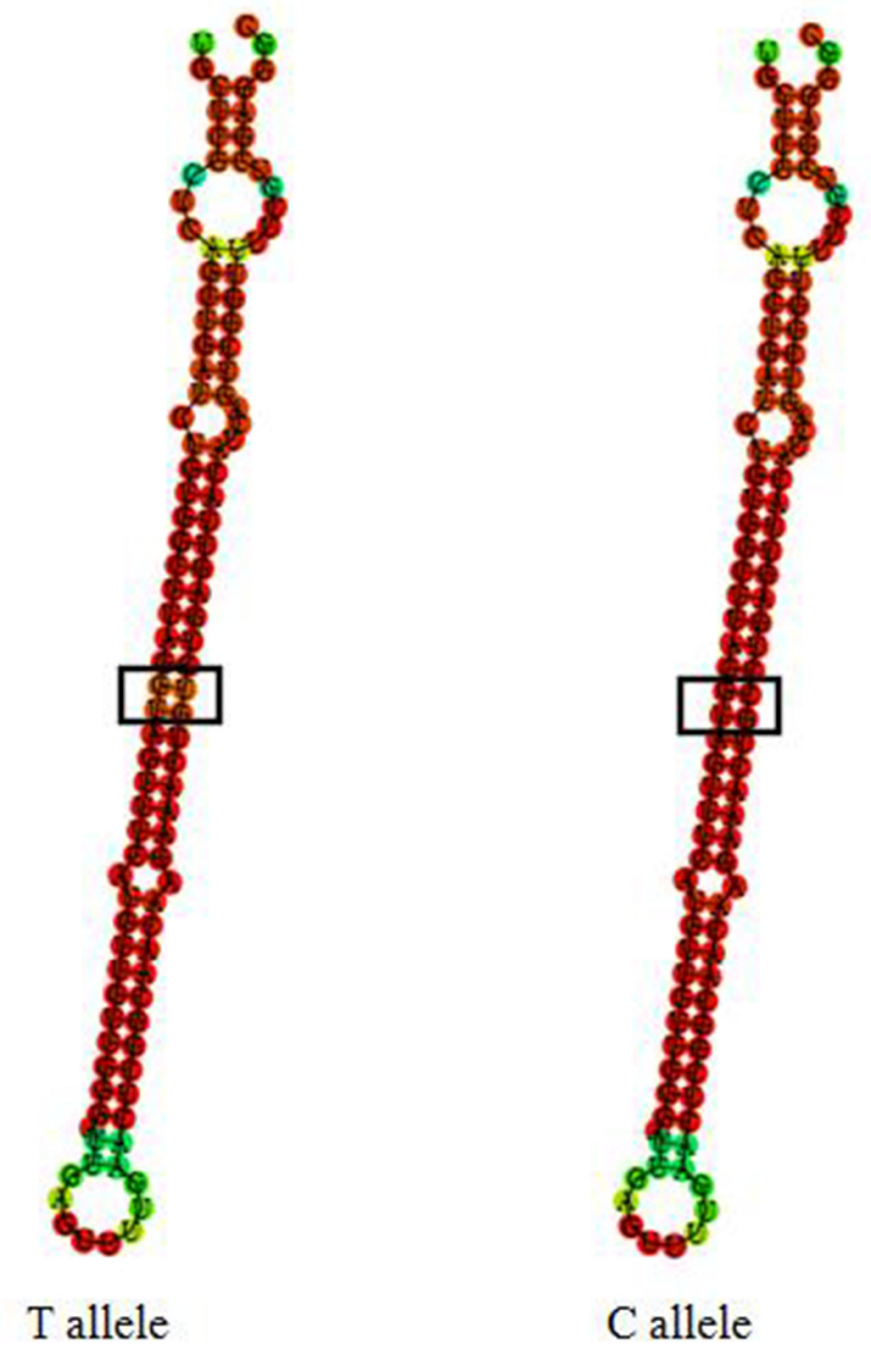
Sequence variations in the miR-196a2 can translate into structural alterations The RNA secondary structure was predicted by RNAHYbrid. Only the most stable secondary structures with the lowest free energy are depicted.

We further tested whether such variants could affect miR-196a2 secondary structure. There was no obvious secondary structure change in T allele compared to C allele from the predicted secondary structures (Figure 2). The optimal free energies were -44.70 Kcal/mol for T allele and -50.30 Kcal/mol for C allele, suggesting a less stable secondary structure for T allele than C allele.

The regulating effect of rs11614913 polymorphism exert on miR-196a expression level was investigated in A549 and H1299 cell lines. QRT-PCR results showed that expression levels of miR-196a precursor increased significantly (350-fold and 230-fold increased respectively) both in H1299 cells transfected with pre-miR-196a-C and pre-miR-196a-T vectors relative to the cells transfected with empty vector (control group), which suggest that the transfection efficiency was acceptable (shown in Supplementary Figure 1). The expression level of mature miR-196a presented no significant differences between cells transfected with expression vectors containing pre-miR-196a2 T and C in H1299 cells as shown in Supplementary Figure 2. In A549 cells transfected with vectors containing pre-miR-196a2 T and C, the expression levels of miR-196a precursor increased 90-fold and 85-fold compared with control group, but there was no statistically significant difference in mature miR-196a expression level between pre-miR-196a2-T and C transfected cells group, results were shown in Supplementary Figures 3 and 4.

## DISCUSSION

This study provided the evidence that common SNP rs11614913 in miR-196a2 might play an important role in lung cancer risk through affecting miR-196a2 expression and secondary structure. The variant genotypes of miR-196a2 rs11614913 were significantly associated with increased risk of lung cancer and miR-196a2 expression. The secondary structure of miR-196a2 with T allele was less stable than that with C allele.

In the present study, we assessed the correlation between miR-196a2 rs11614913 polymorphism and lung cancer risk for Chinese non-smoking females using 1003 incident lung cancer cases and 1003 healthy controls. Our findings suggest that the rs11614913 TC or CC genotypes in miR-196a2 were associated with increased risk of lung cancer and lung adenocarcinoma. There are some inconsistent results about the association between miR-196a2 rs11614913 polymorphism and various malignancies such as breast cancer [20, 21], colorectal cancer [22-25], hepatocellular carcinoma [26-28], gastric cancer [29-30] and lung cancer [16, 31-34].

Because the frequencies of SNPs are significantly different in various populations, it is necessary to study their relationship with the disease in diverse ethnic population. In the present study, the minor allele (C allele) accounted for 52.8% and 46.8% in cases and controls, respectively. To the best of our knowledge, there were only two published studies on the association between miR-196a2 rs11614913 and lung cancer risk in Chinese population and one of them is our published study [16, 31]. Our research group has performed a series of studies in Chinese non-smoking females. Another major strength of our study was the inclusion criteria for the controls, which comprised only healthy subjects who were free of other diseases that might be associated with miRNA SNP, so we could exclude the influence of other diseases on the genotype distribution in the controls. Thirdly, the genotype distribution for rs11614913 was accordant with HWE, further supporting the randomness of our control subjects. Last, the present study included a relatively large number of cases and controls, especially for non-smoking females.

It has also been suggested that miRNA expression levels might be related with the biological and clinical behavior of human tumors [2, 35, 36]. Overall and stratified analysis showed miR-196a2 over-expression in most of the current malignant tumor samples relative to their corresponding cancer-free tissues [37]. The significant increase of miR-196a2 expression was clearly detected in NSCLC compared with paired control tissues [34]. The study about the association between rs11614913 genotypes and miR-196a2 expression showed that carriers of the C allele had significantly higher expression levels of miR-196a2 suggesting that rs11614913 SNP could functionally affect the miR-196a2 expression levels [34, 37, 38]. In this study, we found that there was a significant association between miR-196a2 expression and lung cancer risk. In particular we found a significant association between miR-196a2 CC genotype and high expression, whereas CT or TT genotype showed a very low expression. So we concluded that patients carrying a variant homozygote of miR-196a2 rs11614913 had higher risks of lung cancer possibly through a mechanism of reducing expression of miR-196a2. The present study suggested that rs11614913 might change the secondary structure of miR-196a2 according to the differences of the predicted secondary structures and the optimal free energies between T allele and C allele. However, the evidence is weak because the results were only based on software prediction, without experiment validation.

In our study, the expression level of mature miR-196a-5p didn’t increase in pre-miR-196a2-C and pre-miR-196a2-T transfected cells relative to control group. In process of microRNA maturation, Dicer enzyme play an indispensable role in the process of pre-miRNA to mature microRNA [39]. The expression level of mature miR-196a in our study may result from the reason that the number of microRNA-processing enzyme Dicer in A549 and H1299 cells is limited to process the enormous amount of precursor of microRNA which was transfected into cells. Hu et al. reported that rs11614913 CC was associated with a statistically significant increase in mature hsa-mir-196a expression but not with changes in levels of the precursor, suggesting enhanced processing of the pre-miRNA to its mature form. Furthermore, binding assays revealed that the rs11614913 SNP can affect binding of mature hsa-mir-196a2-3p to its target mRNA [40]. So, the influence of the rs11614913 on the processing of miR-196a maturation may be elucidated in future studies.

Since the SNPs in the regulatory regions attracted much attention in the cancer molecular epidemiology, several recent published results about SNPs in non-coding RNA region have identified the genetic susceptibility factor for the development of cancer [41-43].

In this case-control study, there are still some limitations although we have tried best to reduce the bias. Firstly, this was a hospital-based study and there might be Berkson’s bias. In order to decrease it, we chose the cases and controls from several different hospitals and the controls were healthy subjects obtained from medical examination centers in these hospitals. Secondly, limited information of environmental exposure of study subjects restricted gene-environment interaction analysis. Finally, the current study lacked the functional assessment to determine whether the genetic variants identified in our studies modulate lung cancer risk through their influences on the functions of their target genes. The experiment validation about the effect of rs11614913 on the secondary structure of miR-196a2 is also important to verify the present results. A study comprising possible molecular markers with a larger population would provide further clarification.

In conclusion, our study provides evidence that rs11614913 polymorphism in miR-196a2 might alter individual susceptibility to Chinese female lung cancer through affecting miR-196a2 expression and/or secondary structure. Future larger studies with other ethnic populations and male lung cancer as well as functional tests are required to confirm current findings.

## MATERIALS AND METHODS

### Study subjects

Our research is an ongoing study about impact factors of lung cancer for non-smoking females in Shenyang City, located in northeast China. The females having smoked more than 100 cigarettes in her lifetime was considered as a smoker, otherwise she was defined as a non-smoker. There were 1003 lung cancer patients and 1003 healthy controls (between July 2010 and December 2014), who were all non-smoking females in the present study. The cases and controls were selected according to the inclusion and exclusion criteria, which were the same as reported previously [16]. The informed consents of the study subjects were obtained and the human investigations were approved by the Institutional Review Board of China Medical University. The venous blood of ten milliliter was collected for every participant to detect the SNP.

### Sequence analysis

Genomic DNA sample was extracted by Phenol-chloroform method for each individual. SNP rs11614913 was genotyped by TaqMan allelic discrimination method according to the protocol with a commercially available primer probe set (assay ID C_31185852_10). The three genotypes of rs11614913 were read using the ABI 7500 Fast Sequence Detection System through SDS 4.2.3 software (Applied Biosystems, Lifetechnologies, USA). Quality control was done by randomly selecting 10% of samples to be genotyped twice, and the results were consistent for all of the masked duplicate samples.

### MiRNA expression

In order to determine the expression levels of miRNA, tissues were obtained from patients who had undergone resection for lung cancer. Besides cancer tissues, apparently non-affected tissue was also collected from each patient and used as paired control. In all patients, comparable control tissues were processed for histological examination. Total RNAs were extracted by Trizol method, and then were reverse transcribed with TaqMan Reverse Transcription Kit and assayed by the respective TaqMan microRNA kit (Applied Biosystems, Forster City, CA, USA) according to manufacturer’s instructions. The real time amplification was repeated three times for each sample and the miR-U6B was used as endogenous control. Comparative CT method as well as ΔΔCT and 2^-^ΔΔ^CT^ were used to analyze the miRNA expression level. Real time PCR was performed on ABI 7500 Fast Sequence Detection System (Applied Biosystems, Lifetechnologies, USA). The expression levels of lung cancer tissues and control tissues were compared to study the relationship between miR-196a2 expression and lung cancer risk. Three groups of tissues for three types of rs11614913 SNP were analyzed to evaluate the association between rs11614913 genotypes and the expression of miR-196a2.

### MiRNA secondary structure prediction and optimal free energy calculation

Secondary structures for the wild-type and variant miR-196a2 sequences were predicted using RNAhybrid program (http://bibiserv.techfak.uni-bielefeld.de/rnahybrid/submission.html). The minimum optimal free energies for wild-type and variant sequences also were calculated. The prediction and calculation were carried on according to the instructions and the default setting of the program.

### Cell culture and construction of pre-miR-196a2 expression vector

Human lung adenocarcinoma cells A549 and H1299 cell line were purchased from Shanghai Institute of Biochemistry and Cell Biology, Chinese Academy of Sciences (Shanghai, China) and maintained in RPMI-1640(Biological Industries, Israel) supplemented with 10% fetal bovine serum (Biological Industries, Israel) and 1% penicillin/streptomycin. Expression plasmid vectors GV268 containing neomycin resistance gene, ampicillin resistance gene and whole sequence of precursor of miR-196a2 which was constructed with either wildtype or variant allele of pre-miR-196a2 rs11614913 (pre-miR-196a2 T or C) were purchased from GeneChem (Shanghai, China), empty plasmid vectors GV268 which containing no miR-196a2 precursor sequence were also constructed as negative control (control group).

### Cell transfection, RNA isolation and reverse transcription

A549 cells and H1299 cells were planted on cell culture vessel before transfection of expression plasmid vectors, each vector was transfected into cells using Lipofectamine 3000 transfection reagent (Invitrogen, USA). We use the RNAiso Plus RNA isolation Kit (Takara, Japan) to isolate the total RNA from cells transfected with each of the vectors (empty vector, pre-miR-196a-C, and pre-miR-196a-T) 48 hours after transfection. Instantly, RNA samples were reverse transcribed to cDNA using the MicroRNA First Strand cDNA Synthesis Kit (Sangon, China) which can polyadenylate the miRNAs along with the cDNA synthesis.

### RT-qPCR

To detect the differences of the expression level of miR-196a2 between cells transfected with pre-miR-196a2 T or C expression vectors, quantitative real-time polymerase chain reaction (qRT-PCR) was performed. The specific primers for the miR-196a2 precursor and miRNA-196a2 were as follows: pre-miR-196a2 forward primer: AGCTGATCTGTGGCTTAGGTAG, pre-miR-196a2 reverse primer: CGACGAAAACCGACTGATGT and miR-196a2 forward primer: ATAGGTAGTTTCATGTTGTTGGG. 2xSG Fast qPCR Master Mix (Sangon, China) kit was used for qRT-PCR and U6 was used as internal control. The cDNA was amplified using the primers mentioned above and a universal reverse primer (supplied with MicroRNA First Strand cDNA Synthesis Kit) for detecting the level of mature miR-196a2 and U6 expression. Amplification was measured by SYBR green and fold change of pre-miR-196a and miR-196a expression relative to control group was calculated using the 2-ΔΔCT method normalized to U6. All qRT-PCR reactions were performed in triplicate.

### Statistical analysis

In the case-control study, chi-square test and t-test were used to analyze the categorical and continuous variables, respectively. Hardy-Weinberg equilibrium (HWE) of rs11614913 was examined by the goodness-of-fit χ^2^ test. Unconditional logistic regression was performed to calculate the odds ratios (OR) with their 95% confidence intervals (CI) in order to study the association between the SNP and cancer risk. The mean expression levels of miR-196a2 were analyzed and compared for differences between the wildtype and variant genotypes by the t-test. A statistical significance was defined as P<0.05 and all the statistical tests were 2-sided. All of the analyses were done through SPSS software (Version 20.0, IBM SPSS, Inc. Chicago, IL, USA).

## CONCLUSIONS

The polymorphism rs11614913 in miR-196a2 may be associated with lung cancer risks in Chinese non-smoking females through affecting miR-196a2 expression and secondary structure.

## SUPPLEMENTARY MATERIALS FIGURES


