# Supplementary material for: MiR-196a2 and lung cancer in Chinese non-smoking females: a genetic association study and expression analysis
Source: Oncotarget. 2017 Aug 10;8(41):70890–8. doi: 10.18632/oncotarget.20174 (PMC5642605; doi:10.18632/oncotarget.20174)
Supplement: Supplementary file 1 [file oncotarget-08-70890-s001.pdf]

MiR-196a2 and lung cancer in Chinese non-smoking females: a genetic association study and expression analysis

SUPPLEMENTARY MATERIALS

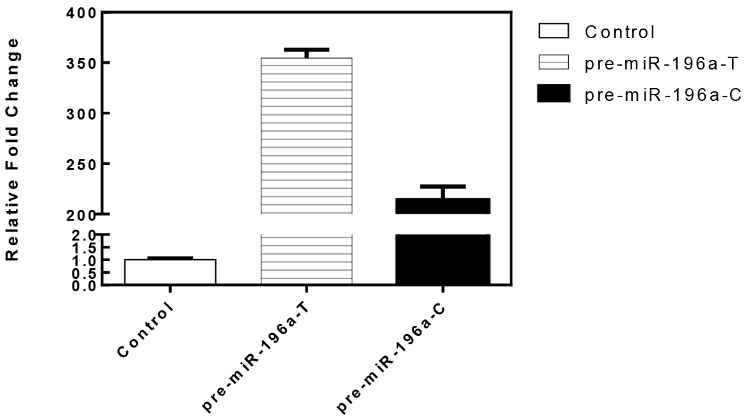

Supplmentary Figure 1: Precursor of microRNA-196a expression in H1299 cells transfected with vectors((empty vector, pre-miR-196a-C, and pre-miR-196a-T).

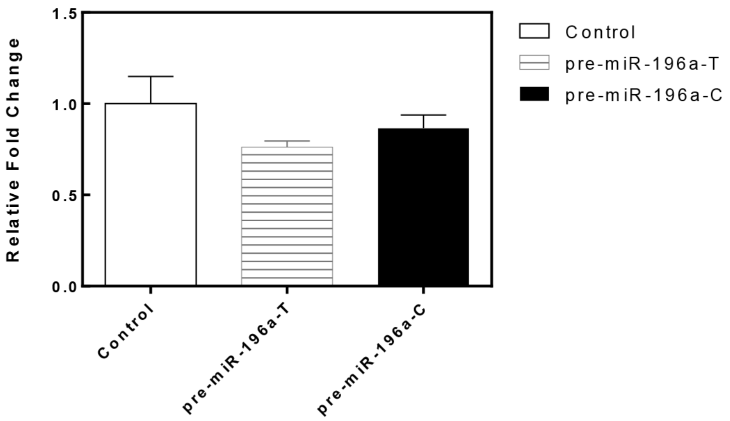

Supplementary Figure 2: MicroRNA-196a-5p expression in H1299 Cells transfected with vectors (empty vector, pre-miR-196a-C, and pre-miR-196a-T).

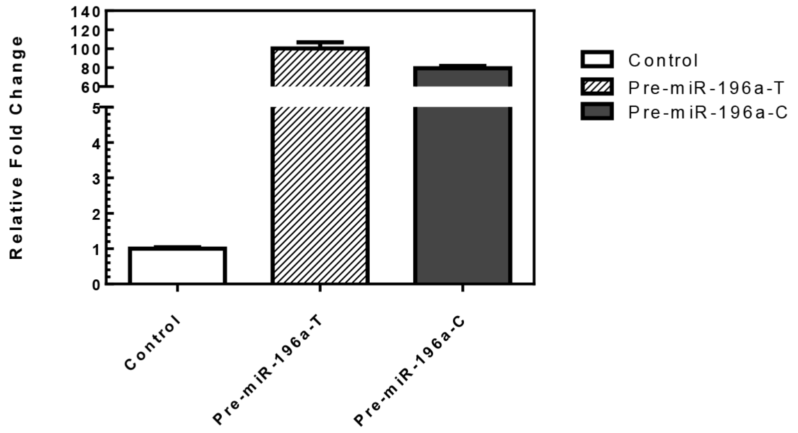

Supplementary Figure 3: Precursor of MicroRNA-196a expression in A549 Cells transfected with vectors (empty vector, pre-miR-196a-C, and pre-miR-196a-T).

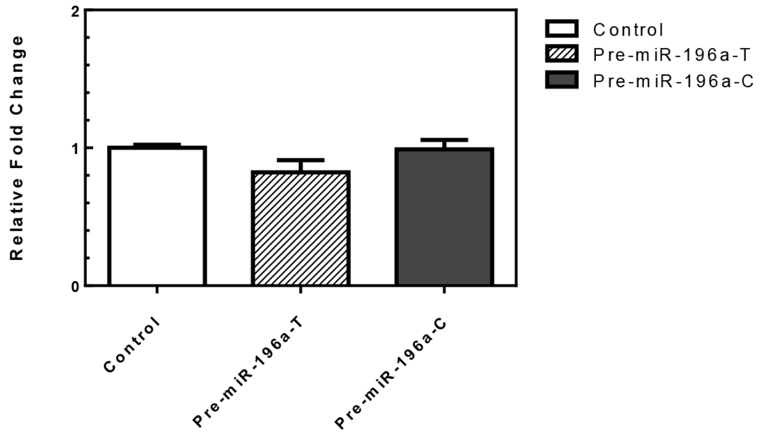

Supplementary Figure 4: MicroRNA-196a-5p expression in A549 Cells transfected with vectors (empty vector, pre-miR-196a-C, and pre-miR-196a-T).
